# Urine miRNA signature as potential non-invasive diagnostic biomarker for Hirschsprung’s disease

**DOI:** 10.3389/fnmol.2024.1504424

**Published:** 2025-01-08

**Authors:** Abhijit Sreepada, Rasul Khasanov, Enas Zoheer Elkrewi, Carolina de la Torre, Judith Felcht, Ahmad A. Al Abdulqader, Richard Martel, Nicolás Andrés Hoyos-Celis, Michael Boettcher, Lucas M. Wessel, Karl-Herbert Schäfer, María Ángeles Tapia-Laliena

**Affiliations:** ^1^Translational Medical Research/International Master in Innovative Medicine Master Program, Medical Faculty of Mannheim, University of Heidelberg, Mannheim, Germany; ^2^Department of Psychiatry and Psychotherapy, Central Institute of Mental Health, Medical Faculty Mannheim, Heidelberg University, Mannheim, Germany; ^3^Department of Pediatric Surgery, Medical Faculty of Mannheim, University of Heidelberg, Mannheim, Germany; ^4^NGS Core Facility, Medical Faculty Mannheim, University of Heidelberg, Mannheim, Germany; ^5^Department of Surgery, College of Medicine, King Faisal University, Al Hofuf, Saudi Arabia; ^6^Working Group Enteric Nervous Systems (AGENS), University of Applied Sciences Kaiserslautern, Campus Zweibrücken, Kaiserslautern, Germany

**Keywords:** Hirschsprung’s disease (HSCR), enteric nervous system (ENS), microRNA (miRNA), non-invasive diagnostic, urine extracellular vesicles

## Abstract

Hirschsprung’s disease (HSCR) is characterized by congenital absence of ganglion cells in the gastrointestinal tract, which leads to impaired defecation, constipation and intestinal obstruction. The current diagnosis of HSCR is based on Rectal Suction Biopsies (RSBs), which could be complex in newborns. Occasionally, there is a delay in diagnosis that can increase the risk of clinical complications. Consequently, there is room for new non-invasive diagnostic methods that are objective, more logistically feasible and also deliver a far earlier base for a potential surgical intervention. In recent years, microRNA (miRNA) has come into the focus as a relevant early marker that could provide more insights into the etiology and progression of diseases. Therefore, in the search of a non-invasive HSCR biomarker, we analyzed miRNA expression in urine samples of HSCR patients. Results from 5 HSCR patients using microarrays, revealed hsa-miR-378 h, hsa-miR-210-5p, hsa-miR-6876-3p, hsa-miR-634 and hsa-miR-6883-3p as the most upregulated miRNAs; while hsa-miR-4443, hsa-miR-22-3p, hsa-miR-4732-5p, hsa-miR-3187-5p, and hsa-miR-371b-5p where the most downregulated miRNAs. Further search in miRNAwalk and miRDB databases showed that certainly most of these dysregulated miRNAs identified target HSCR associated genes, such as *RET, GDNF, BDNF, EDN3, EDNRB, ERBB, NRG1, SOX10;* and other genes implied in neuronal migration and neurogenesis. Finally, we could also validate some of these miRNA changes in HSCR urine by RT-qPCR. Altogether, our analyzed HSCR cohort presents a dysregulated miRNA expression presents that can be detected in urine. Our findings open the possibility of using specific urine miRNA signatures as non-invasive HSCR diagnosis method in the future.

## 1 Introduction

Hirschsprung’s disease (HSCR) (incidence 1/5,000 births) is a congenital gastrointestinal disorder caused by aganglionosis of the distal colon, which in newborns causes impaired defecation, constipation, and intestinal obstruction due to a lack of relaxation (Heuckeroth, 2018). The latter can impact either only short segments, the whole colon, and in few cases the whole gut (Heuckeroth, 2018). Currently, the international evidence-based treatment is the surgical removal of the aganglionic bowel. However, after undergoing surgical resection patients may have a restricted quality of life, i.e., due to recurrent enterocolitis (Menezes and Puri, 2006), partial incontinence or persisting defecation problems (Calkins, 2018).

Although HSCR anatomy is well described, individual phenotypes are complex, often making the diagnosis difficult (Heuckeroth, 2018). The most reliable diagnosis method for HSCR diagnosis include methods such as Rectal Suction Biopsies (RSB) that include the submucous layer (Lewis et al., 2003; de Lorijn et al., 2006; Romero et al., 2024). Usually, RSBs are combined with acetylcholinesterase (AChE) staining. This habitually requires high quality thick tissue and several biopsies in order to confirm the absence of ganglion cells, which sometimes leads to clinical complications (Friedmacher and Puri, 2015; Neeser et al., 2024). Nonetheless, it is difficult to decipher the functionality of potential immature ganglia in newborns, where an experienced pathologist together with a good clinical team could be required for a correct, unambiguous HSCR diagnosis. Consequently, the complexity and potential human errors can further exacerbate the risk of erroneous diagnoses in complicated cases (Shayan et al., 2004; Erbersdobler, 2024).

Recent studies have attempted to improve RBSs-AChE’s diagnosis precision. Methods such as software-based quantification of the AChE-stained cholinergic hyperinnervation (Braun et al., 2024), or measuring neural fiber trunk diameter for precisely establishing the length of the aganglionic segment in patients (Talebi et al., 2024) have been described. Yet, the requirement of good quality RBSs for the AChE staining continues to remain the key-challenge limiting their applicability.

Calretinin IHC has emerged as an alternative, promising form of staining. It presents several advantages in comparison to AChE histochemistry, like the possibility to perform the test on paraffin-embedded tissue sections (AChE requires cryosections), a straightforward staining pattern of clear binary interpretation (negative or positive), cost-effectiveness, and being performable regardless of patient age (Muller et al., 2015; Romero et al., 2024). This can provide an earlier HSCR diagnosis than AChE, and help to avoid repeated RSBs. This would allow patients to undergo surgery in the first few months of life, thereby reducing the number of enterostomies and posterior complications (Romero et al., 2024). Nevertheless, high quality RSBs are still needed, and many can be inconclusive even using Calretinin staining (Korsager et al., 2023).

Despite their advantages, all these tests need highly specialized equipment, radiation exposure, additional hospital time and are challenging to perform in neonates (Osatakul et al., 1999; Diamond et al., 2007). Given the importance of an early diagnosis, there is thus a room for novel diagnostic techniques that are non-invasive, are relatively accessible to perform, and are easy to interpret, thus allowing to decide whether further biopsies are necessary or could be avoided.

Posttranscriptional regulation by microRNAs (miRNAs), small non-coding RNA molecules of 19–25 nucleotides, which constrain the expression of target genes by directly binding to their mRNAs, is an important regulatory mechanism of gene expression (Hosako et al., 2009). Usually, miRNAs are secreted from most cell types inside exosomal extracellular vesicles, which keep miRNA sequences stable (Mall et al., 2013). These vesicles and miRNAs can later be specifically detected in body fluids such as urine, blood, cerebrospinal fluid or saliva (Kroh et al., 2010; O’Brien et al., 2018; Salehi and Sharifi, 2018).

Given their ubiquitous presence and their associations with health and disease, in recent years, miRNAs have become popular as biomarkers, which refer to biological, objective markers representative of certain healthy or diseased states (Wang et al., 2016; Condrat et al., 2020). Some of the first published studies testing the diagnostic utility of miRNA in cancer were already published in around 2008 (Lawrie et al., 2008; Mitchell et al., 2008). Both studies used circulating miRNA in serum or plasma, which was a relatively non-invasive and feasible technique. Today there are multiple published studies that report the role of circulating miRNA as a diagnostic marker across several different types of cancers (Chen et al., 2008; Liu et al., 2012; Bertoli et al., 2015; Subramani et al., 2015; Fabris et al., 2016), cardiovascular diseases (Zhou et al., 2018), sepsis (Benz et al., 2016), gestational diabetes mellitus (Zhao et al., 2011), Parkinson’s disease (Gries et al., 2021) and several other diseases.

Nevertheless, it was the presence of specific urine miRNAs described in other diseases, like nephrotic syndrome (Luo et al., 2013), prostate cancer (Korzeniewski et al., 2015) or to determine exposure to pesticides (Weldon et al., 2016), that led to our hypothesis that similar specific miRNA patterns probably also exist in urine of HSCR patients. Indeed, some studies have already identified dysregulated miRNA expression in intestinal tissue (Shen et al., 2016; Zhang et al., 2024) or serum (Tang et al., 2014) from HSCR patients.

However, bearing in mind the age of HSCR patients, we designed this “proof of concept” study in urine with the aim of identifying a non-invasive biomarker to complement the histology and clinical diagnosis in the future.

## 2 Materials and methods

### 2.1 Patient samples

The collection and use of patient material was performed according to informed consent signed by patients’ parents and approved by the “Medizinische Ethik-Kommission II” of the Medical Faculty Mannheim, University of Heidelberg (2011-237 N-MA). Samples were only identified by sequential code numbers with no other identifying details.

Urine from 5 patients diagnosed with HSCR and 5 healthy controls was collected “clean-catched” and stored at −80°C until analysis. The characteristics of the patient’s cohort is described in Supplementary Table S1.

### 2.2 miRNA extraction from urine

miRNA was obtained using the extracellular vesicle extraction process. Urine samples were pre clarified by spinning for 15 min. at 3000 rpm. To isolate the extracellular vesicles, the supernatants were again ultra-centrifuged for 1 h at 28,000 g. Following this, the supernatant was removed, and the pellet was resuspended in TRIzol™ Reagent (Invitrogen, Thermofisher Scientific Inc., Waltham, MA, United States) to isolate RNA according to manufacturer’s protocol. Following this, the mirVana™ miRNA Isolation Kit (Invitrogen, Thermofisher Scientific Inc., Waltham, MA, United States) was utilized for the rest of the miRNA purification, also according to manufacturer’s protocol.

The miRNA concentration was measured with the infinite M200 micro plate reader (Tecan, Mainz-Kastel, Germany) and the miRNA quality was tested with the RNA 6000 Nano Kit (Agilent Technologies, Santa Clara, CA, United States) and the Agilent Bioanalyzer 2,100 (Agilent Technologies Inc., Santa Clara, CA, United States).

### 2.3 miRNA microarrays

miRNA expression profiling was performed using the GeneChip™ miRNA 4.0 Arrays (Thermo Fisher Scientific Inc., Waltham, MA, United States) with the help of our NGS Core Facility (Medical Faculty Mannheim, University of Heidelberg, Mannheim, Germany). Biotinylated antisense cDNA was prepared according to the standard labeling protocol with the GeneChip^®^ WT Plus Reagent Kit and the GeneChip^®^ Hybridization, Wash and Stain Kit (both from Thermo Fisher Scientific Inc., Waltham, MA, United States). Afterwards, the hybridization on the chip was performed on a GeneChip Hybridization oven 640, then dyed in the GeneChip Fluidics Station 450 and thereafter scanned with a GeneChip Scanner 3,000.

Data was analyzed using a commercial software package SAS JMP15 Genomics. The raw fluorescence intensity values were normalized applying quantile normalization and RMA background correction. Thereafter, One Way ANOVA was performed to identify differential expressed genes. miRNAs with an adjusted *p*-value ≤0.05 were considered as significant.

The raw and normalized data are deposited in the Gene Expression Omnibus database (accession No. GSE277874).^1^

### 2.4 miRNA selection and database target search

Data was sorted through, and 10 miRNAs (*p*-value ≤0.05) were selected based on their expression fold: the 5 that were most upregulated, and the 5 that were most down-regulated.

Based on this, the corresponding targets of each miRNA were searched using the miRWalk database (http://mirwalk.umm.uni-heidelberg.de/) (Sticht et al., 2018). First, the putative target list of each of the 10 selected miRNAs was downloaded, where the first top 10 targeted genes were listed. The resulting miRWalk list was cross-referenced with a second database, miRDB (mirdb.org) (Chen and Wang, 2020).

Additionally, already described HSCR-related genes (*BDNF, ECE1*, *EDNRB, EDN3 and ERBB3, GDNF, RET, NRG1, SOX10,* etc.) were searched in both databases for all the miRNAs. Ultimately, other neuronal- related genes (f.i.: linked to neuronal migration, neuronal crest development, neurogenesis, etc.) were also browsed in the same databases.

### 2.5 RT-qPCR

cDNA synthesis was performed using the TaqMan™ MicroRNA Reverse Transcription Kit (Applied Biosystems Inc., Thermo Fisher Scientific Inc., Waltham, MA, United States) according to manufacturer’s instructions. The reaction was carried out using a peqSTAR Thermocycler (PeqLab Biotechnology GmbH, Erlangen, Germany) as follows: 5 min. Denaturation at 70°C, 10 min. Annealing at 20°C, 60 min. Elongation at 40°C, and 10 min. Inactivation at 70°C.

The Sensifast SYBR Low-ROX Kit (BIO-94020, *Bioline,* Meridian Biosciences, OH, United States) was used for the RT-qPCR following the manufacturer’s instructions. Briefly, amplification reactions were run using a T_m_ of 57°C, in the QuantStudio 5 device (Applied Biosystems Inc., Thermo Fisher Scientific Inc., Waltham, MA, United States) as follows: Hold stage: *Step 1*: 2 min. at 50°C, *Step 2 (denaturation):* 10 min. at 95°C; PCR Stage (40 cycles): *Step 1 (denaturation):* 15 s at 95°C, *Step 2 (annealing):* 1 min. at 57°C; followed by a final Melting Curve Stage: *Step 1*: 15 s at 95°C, *Step 2:* 1 min. at 57°C, *Step 3 (Dissociation)*: 15 s at 95°C.

Primers’ sequences were purchased to OriGene Technologies GmbH, Herford, Germany. In total, 3 housekeeping control primers and 10 miRNA primers were used (see Supplementary Table S2).

The comparative 2^−ΔΔCt^ method was used to calculate gene expression, where data were first normalized to the housekeeping standard (dCt: Target Ct—Housekeeping Ct). Then, for each gene sample ddCt (ddCt: Sample dCt—Calibrator dCt) was calculated using the average of the controls as a calibrator. Finally, fold 2^−ΔΔCt^ was calculated for each miRNA.

### 2.6 Statistical analysis

Statistical analysis was performed using the One Way ANOVA method for the miRNA microarray data analysis. The F.N. Test was used to compare differences in miRNA expression between controls and HSCR patients in the RT-qPCR test. Differences were considered statistically significant at *p*-value ≤0.05.

## 3 Results

### 3.1 miRNA microarray analysis of HSCR urine

In an initial “proof of concept” study, urinary miRNA from 5 HSCR patients and 5 healthy controls was analyzed using the GeneChip™ miRNA 4.0 Arrays (Thermo Fisher Scientific Inc., Waltham, MA, United States). The original array data is available on the NCBI Gene Expression Omnibus (GEO) browser (Reference number GSE277874).

**Figure 1:**
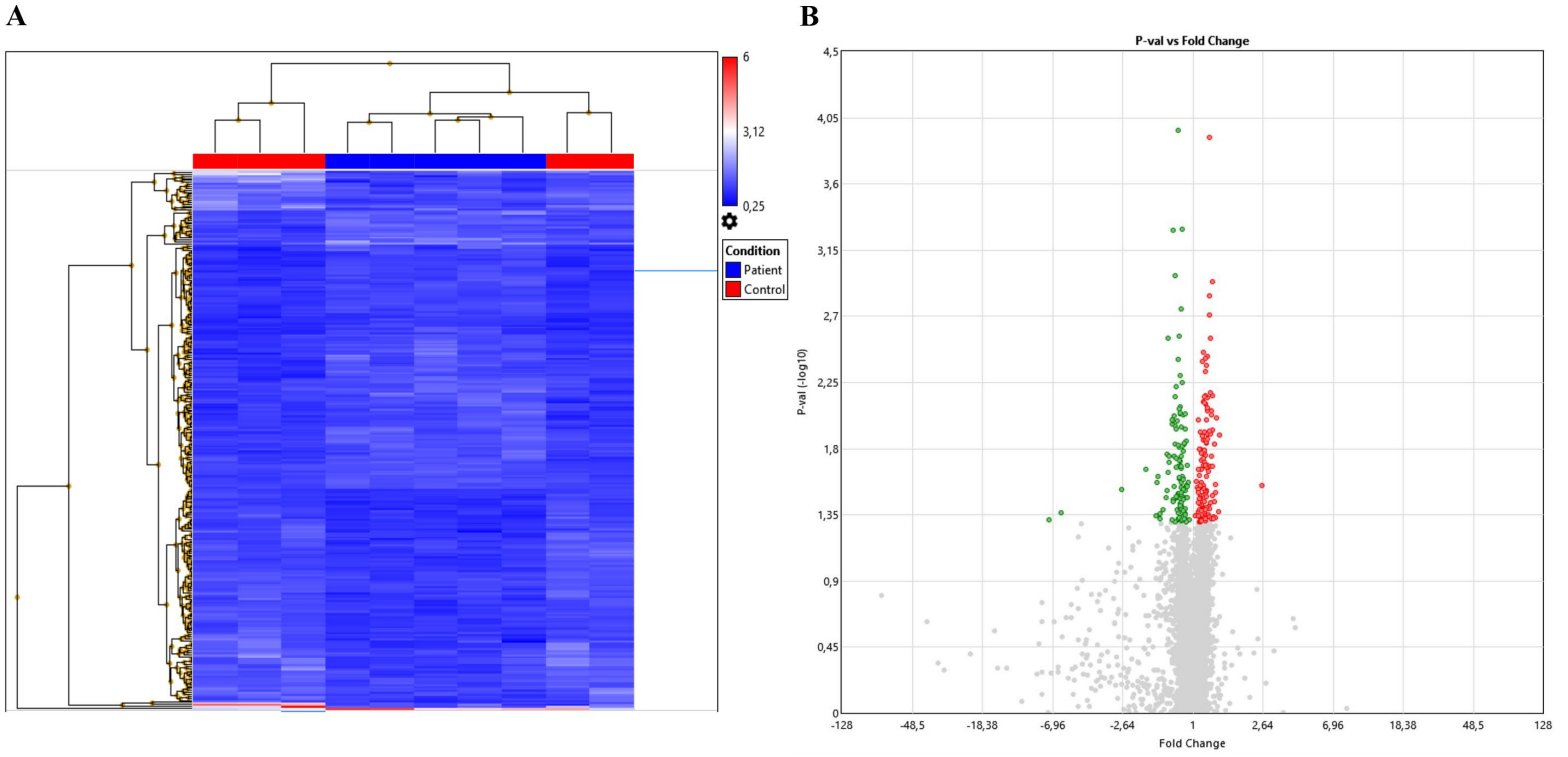
Overview of microarray analysis. **(A)** Heatmap of relative miRNAs expression. Differential expression is shown between Controls and HSCR samples, color scale represents up- (red), no threshold (gray), or downregulation (blue). **(B)** Volcano plot showing miRNAs distribution after ANOVA analysis.

Despite the limited size of our studied cohort, we could find significant differences in the expression of some urinary miRNAs between controls and HSCR patients. Though still preliminary results, they may be used as a basis for further research. The heatmap in Figure 1 shows the results of the clustering analysis based on the normalized expression values of up- (red) and down-regulated (blue) miRNAs (Figure 1). The distribution of the miRNAs is represented in the Volcano plot (Figure 1).

After statistical analysis, data was sorted out based in fold changes in order to select the miRNAs that were most dysregulated. Results showed hsa-miR-378 h, hsa-miR-210-5p, hsa-miR-6876-3p, hsa-miR-634 and hsa-miR-6883-3p as the most upregulated miRNAs; while hsa-miR-4443, hsa-miR-22-3p, hsa-miR-4732-5p, hsa-miR-3187-5p, and hsa-miR-371b-5p were the most downregulated miRNAs in urine of HSCR patients compared to urine of healthy controls (*p*-value ≤0.05) (see Table 1).

**Table 1:** List of the 10 selected most dysregulated miRNAs identified in HSCR urine.

	ID	Fold change	P-val	Probe set name	Transcript ID
Upregulated	20518842	2.62	0.03	MIMAT0018984_st	hsa-miR-378 h
	20500464	1.45	0.01	MIMAT0026475_st	hsa-miR-210-5p
	20525714	1.39	0.01	MIMAT0027653_st	hsa-miR-6876-3p
	20504387	1.36	0.03	MIMAT0003304_st	hsa-miR-634
	20525728	1.34	0.01	MIMAT0027667_st	hsa-miR-6883-3p
Downregulated	20518818	−7.3	0.05	MIMAT0018961_st	hsa-miR-4443
	20500144	−6.19	0.04	MIMAT0000077_st	hsa-miR-22-3p
	20519576	−2.68	0.03	MIMAT0019855_st	hsa-miR-4732-5p
	20515622	−1.92	0.02	MIMAT0019216_st	hsa-miR-3187-5p
	20519615	−1.66	0.05	MIMAT0019892_st	hsa-miR-371b-5p

### 3.2 Most dysregulated urine miRNAs in HSCR patients target genes associated to HSCR pathology

With the aim of verifying the potential role of those miRNAs in HSCR development, we performed a target analysis exploration with the help of miRWalk (Sticht et al., 2018) and miRDB (Chen and Wang, 2020) databases.

Initially, we investigated the top 10 targeted genes in both databases with the highest score (see Table 2). Despite the high diversity of genes targeted by the selected miRNAs, it was possible to already find genes like *ALKBH5*, which was related to enteric neuronal crest migration in HSCR (Wang et al., 2021), or genes linked to neuronal migration, like *DAB2IP* (Lee et al., 2012) or *NF1* (Sanchez-Ortiz et al., 2014), as well as to neuronal motility, like *GMR5* (Turunen et al., 2018), or to other neuronal processes such as *BTG2* (el-Ghissassi et al., 2002) or *KIRREL3* (Hisaoka et al., 2021).

**Table 2:** Target database analysis of the selected dysregulated miRNAS in HSCR urine.

		Database					
		miRWalk		miRDB		miRWalk + miRDB	
	miRNA	Gene symbol	Score	Gene symbol	Score	Gene symbol	Score
Upregulated	hsa-miR-378 h	SLC12A6	1.00	KLK4	98	ERAP1	1.00
		NF1	1.00	NR2C2	98	BAALC	1.00
		CARHSP1	1.00	NKX3-1	97	PODXL	1.00
		MLPH	1.00	KIAA1522	97	KCNMA1	1.00
		TRAPPC5	1.00	PHC3	96	SHE	1.00
		FILIP1L	1.00	ELAC1	92	TSPAN17	1.00
		BPIFA3	1.00	ZNF124	92	PRLR	1.00
		ST3GAL5	1.00	RAB10	91	GRIK3	1.00
		SLC16A3	1.00	NCAPG	91	APOC2	1.00
		RUFY2	1.00	KCNIP2	91	BRD4	1.00
	hsa-miR-210-5p	SLC12A6	1.00	BTG2	99	TMEM80	1.00
		SMIM8	1.00	SH3BP4	96	FAM131B	1.00
		FRMD6	1.00	BICD2	95	AP2B1	1.00
		CEP85L	1.00	ZNF385B	95	LDLRAD2	1.00
		ZNF565	1.00	FAM161A	94	PRAMEF6	1.00
		ABHD12	1.00	EP300	94	ATP7B	1.00
		MLPH	1.00	TENM2	93	PKP1	1.00
		TMEM80	1.00	ANKRD13B	93	ATP2B3	1.00
		ZNF205	1.00	KCNAB2	91	SLC11A1	1.00
		C10orf53	1.00	KIRREL1	91	PKP1	1.00
	hsa-miR-6876-3p	DLGAP4	1.00	PAPOLB	99	SLAIN1	1.00
		NUPR1	1.00	CDH13	96	SIRPA	1.00
		FRMD6	1.00	BCL11A	95	RBFOX2	1.00
		CEP85L	1.00	DPY19L3	95	KIAA1755	1.00
		ACBD5	1.00	SLAMF7	95	ARGFX	1.00
		MLPH	1.00	RNF6	94	CENPP	1.00
		TMEM80	1.00	DUSP4	94	SLC7A2	1.00
		TRAPPC5	1.00	SMCP	93	RAB37	1.00
		FILIP1L	1.00	PIAS2	93	GPR179	1.00
		SLC5A10	1.00	TIPARP	93	MITF	1.00
	hsa-miR-634	SLC35A2	1.00	ARGLU1	98	PPP4R1	1.00
		NF1	1.00	CRISPLD2	98	HEY1	1.00
		TPK1	1.00	FBXW7	97	METTL17	1.00
		ABHD12	1.00	HMGXB4	96	ABI1	1.00
		TRAPPC5	1.00	CLIC5	96	NAA30	1.00
		CYLD	1.00	NRXN3	95	ENAH	1.00
		PPP4R1	1.00	FAM3D	94	UACA	1.00
		KIAA0895L	1.00	IGDCC4	94	IRGQ	1.00
		SYPL2	1.00	OAZ1	94	HMGXB4	1.00
		HEY1	1.00	ATP6V0B	93	ATXN7	1.00
	hsa-miR-6883-3p	SLC35A2	1.00	MEX3D	98	TENT4B	1.00
		NF1	1.00	GRIN2A	97	PAQR3	1.00
		DLGAP4	1.00	NCAPG2	96	SCN3B	1.00
		NUPR1	1.00	GID8	95	ATP6V1C2	1.00
		ACBD5	1.00	ARFGEF1	95	C18orf32	1.00
		TMEM80	1.00	OXR1	95	MYCL	1.00
		TRAPPC5	1.00	HECA	94	AIPL1	1.00
		SLC5A10	1.00	ZFX	94	DCAF4L1	1.00
		C10orf53	1.00	SUMF1	94	RAB3IP	1.00
		SLC16A3	1.00	LRP2	93	RAP1B	1.00
Downregulated	hsa-miR-4443	SLC35A2	1.00	KDM2A	98	SUMF2	1.00
		GSDMB	1.00	PTPRJ	97	GLIS3	1.00
		SUMF2	1.00	PIK3C2B	97	PEG10	1.00
		TRAPPC5	1.00	KRTAP4-12	97	SGSM1	1.00
		TGFB1I1	1.00	AGPAT4	97	ACBD7	1.00
		SLC5A10	1.00	RGS7	96	TYW5	1.00
		CAST	1.00	MOGAT3	95	KREMEN1	1.00
		ZNF205	1.00	EBF3	95	FER1L6	1.00
		C10orf53	1.00	EIF4G3	95	KCNIP1	1.00
		NSD2	1.00	ST3GAL1	95	RBFOX2	1.00
	hsa-miR-22-3p	ST8SIA2	1.00	GRM5	100	C17orf58	1.00
		TLK2	1.00	FUT9	99	NEFM	1.00
		CYB561	1.00	ESR1	97	MPZL3	1.00
		DDX25	1.00	ELOVL6	97	COA7	1.00
		TTYH2	1.00	EMILIN3	97	NPAS3	1.00
		PIWIL2	1.00	LAMC1	97	MAT2A	1.00
		B3GNT4	1.00	NET1	97	NPNT	1.00
		HMBOX1	1.00	PDSS1	96	TYRO3	1.00
		CENPX	1.00	RCOR1	96	COA7	1.00
		RPGRIP1L	1.00	DDIT4	96	AGBL5	1.00
	hsa-miR-4732-5p	SLC12A6	1.00	ARSE	90	CDC42SE2	1.00
		SMIM8	1.00	AMMECR1	90	VPS26A	1.00
		NF1	1.00	ALKBH5	90	CALML4	1.00
		DLGAP4	1.00	KLLN	89	FAM13A	1.00
		NUPR1	1.00	TAF9B	89	DGKK	1.00
		GEMIN8	1.00	WASL	88	ZNF766	1.00
		CARHSP1	1.00	ANXA7	87	WDR33	1.00
		CEP85L	1.00	PSMA5	87	GABRB3	1.00
		ACBD5	1.00	FAM222B	86	STS	1.00
		SUMF2	1.00	KIRREL3	86	LUC7L3	1.00
	hsa-miR-3187-5p	SLC12A6	1.00	TMEM41B	98	CEP44	1.00
		NUPR1	1.00	AHI1	97	FOXL2NB	1.00
		FRMD6	1.00	DAB2IP	97	SLC4A8	1.00
		ZNF565	1.00	BTRC	96	CBFA2T2	1.00
		ACBD5	1.00	FMO5	96	SCNN1G	1.00
		SUMF2	1.00	SIKE1	96	KCNIP1	1.00
		MLPH	1.00	SAR1A	95	CBFA2T2	1.00
		TMEM80	1.00	SEC63	95	PCARE	1.00
		TRAPPC5	1.00	YTHDF1	95	SC5D	1.00
		STK26	1.00	ARRDC3	94	SH3PXD2B	1.00
	hsa-miR-371b-5p	DDX60L	1.00	ZNF845	100	MINDY2	1.00
		ZDHHC3	1.00	ZC3H6	100	NKAIN2	1.00
		HPS4	1.00	SPAG9	100	ZNF765	1.00
		PRKCH	1.00	SLC35E2A	100	GLUL	1.00
		TCF7L2	1.00	ZNF850	100	CRELD1	1.00
		MARF1	1.00	AKR1D1	100	ARHGAP5	1.00
		MEF2D	1.00	TMEM33	100	CELF2	1.00
		PRMT2	1.00	ZBTB3	100	TNRC6B	1.00
		GID8	1.00	S1PR2	100	TMCC1	1.00
		REL	1.00	UQCC3	100	DCUN1D2	1.00

Further analysis in the same databases revealed that most of the dysregulated miRNAs in HSCR urine identified in the microarray regulate genes strongly associated to HSCR (see Table 3). For instance, genes like *EDNRB* (Puffenberger et al., 1994), *RET* (Luo et al., 1993; Emison et al., 2005), *BDNF* (Schriemer et al., 2016), *GDNF* (Parisi and Kapur, 2000), *SOX10* (Prasad et al., 2011) etc. are targets of many of our detected miRNAs. In addition, a wide spectrum of different genes related to HSCR (Parisi and Kapur, 2000; Heuckeroth and Schäfer, 2016; Luzón-Toro et al., 2020) such as *EDN3*, *ECE-1*, *ERBB3* (Gershon, 2021), *NRG1* (Gui et al., 2013), *NTKR3* (Sanchez-Mejias et al., 2009) or *L1CAM* (Jackson et al., 2009), have been also found in the databases (Table 3). Aside from those, other genes participating in signaling processes, like *MAPK8* (Kuil et al., 2021), *TMP3* (Camilleri et al., 2019) or *PIK3C2B* (Fu et al., 2020) have also recently been connected to ENS formation and HSCR.

**Table 3:** Specific miRNA targets associated with HSCR or neuronal processes (neuronal migration, neurogenesis, etc.).

		Database									
		miRWalk		miRWalk			miRDB				
		HSCR-rel. genes		Neuronal-rel. Genes			HSCR-rel. Genes		Neuronal-rel. Genes		
	miRNA	Gene symbol	Score	Gene symbol	Score	References.	Gene symbol	Score	Genesymbol	Score	References
Upregulated	hsa-miR-378 h	GDNF	1.00	NF2	1.00	Toledo et al. (2018)			NPAS4	85	Kwon et al. (2024)
		NGR1	1.00	NFASC	1.00	Zonta et al. (2008)			NEUROD4	71	
		EGFR	0.92	TPM3	1.00	Camilleri et al. (2019)			NEUROD1	63	Singh et al. (2022)
				MAPK8	0.92	Kuil et al. (2021)					
				NEGR1	0.92	Kim et al. (2014)					
	hsa-miR-210-5p	RET	1.00	ALKBH5	1.00	Wang et al. (2021)	SOX10	76	BTG2	99	
		L1CAM	1.00	MAPK8	1.00	Kuil et al. (2021)			BICD2	95	Tsai et al. (2020)
		GDNF	0.92	NFASC	0.92	Zonta et al. (2008)			KIRREL1	91	
		BDNF	0.85	BTG2	0.92	el-Ghissassi et al. (2002)			RCOR2	50	
		SOX10	0.85	NEGR1	0.92						
	hsa-miR-6876-3p	ERBB3	1.00	BCL11A	1.00	Wiegreffe et al. (2015)	NRG1	60	BCL11A	95	Wiegreffe et al. (2015)
		GDNF	1.00	MAPK8	1.00	Kuil et al. (2021)			MAPK8	76	Kuil et al. (2021)
		EDNRB	1.00	NTM	0,96				NETO1	71	
		ECE1	1.00	NEGR1	0.92	Kim et al. (2014)			NEGR1	70	Kim et al. (2014)
		BDNF	0.92	NFASC	0.92	Zonta et al. (2008)			NPAS3	62	Liu et al. (2022)
				RELA	0.92	Elkrewi et al. (2024)			NTM	54	Salluzzo et al. (2023)
	hsa-miR-634	ERBB3	0.92	NRXN3	1.00	Harkin et al. (2019)	ERBB4	52	FBXW7	97	Jandke et al. (2011)
		GDNF	0.85	RELA	1.00	Elkrewi et al. (2024)			NFASC	66	Zonta et al. (2008)
		ECE1	0.85	ALKBH5	0.92	Wang et al. (2021)			NEUROG2	60	Heng et al. (2008)
				MANF	0.92	Tseng et al. (2017)			MANF	56	Tseng et al. (2017)
				RELB	0.92	Denis-Donini et al. (2005)			NAV3	55	
	hsa-miR-6883-3p	BDNF	1.00	MAPK8	1.00	Kuil et al. (2021)			CALN1	61	
		GDNF	0.92	NFASC	0.98	Zonta et al. (2008)			ARFGEF1	95	You et al. (2022)
		RET	0.85	NEGR1	0.92	Kim et al. (2014)			MAPK8	69	Kuil et al. (2021)
		EDNR3	0.85	NPAS3	0.92	Liu et al. (2022)					
		EDNRB	0.85	RELA	0.88	Elkrewi et al. (2024)					
				CALN1	61						
Downregulated	hsa-miR-4443	GDNF	1.00	CALN1	1.00		PIK3C2B	92	EBF3	95	Friocourt (2011)
		NRG1	0.92	NF2	1.00	Toledo et al. (2018)	NTRK3	85	NTRK2	84	
		ERBB3	0.85	NEGR1	0.92	Kim et al. (2014)	ECE1	70	NFASC	66	
		ECE1	0.85	NEUROG2	0.92	Heng et al. (2008)			CALN1	64	
		SOX10	0.85	NFASC	0.92				NDEL1	64	
				MAPK8	0.88	Kuil et al. (2021)			PACSIN1	61	
	hsa-miR-22-3p	NRG1	1.00	NFASC	1.00		ERBB3	95	GRM5	100	Turunen et al. (2018)
		EDN3	0.85	NF2	1.00	Toledo et al. (2018)	ERBB4	54	NET1	97	Robinson et al. (2021)
		BDNF	0.92	GRM5	1.00	Turunen et al. (2018)			RCOR1	97	Mandel et al. (2011)
				RELA	1.00	Elkrewi et al. (2024)			PHF8	96	Riveiro et al. (2017)
				NEGR1	0.95	Kim et al. (2014)			NEGR1	60	
	hsa-miR-4732-5p	BDNF	1.00	MAPK8	1.00	Kuil et al. (2021)	RET	61	ALKBH5	95	Wang et al. (2021)
		GDNF	1.00	PHF8	1.00	Riveiro et al. (2017)	GDNF	55	TAF9B	89	Herrera et al. (2014)
		SOX10	1.00	TAF9B	0.96	Herrera et al. (2014)	EDN3	65	ANXA7	87	Rick et al. (2005)
		L1CAM	1.00	BCL11A	0.92	Wiegreffe et al. (2015)			KIRREL3	86	Hisaoka et al. (2021)
		NRG1	1.00	NEUROG2	0.92	Heng et al. (2008)					
		ECE1	0.92	NFASC	0.92						
		ERBB3	0.92	NF2	0.92	Toledo et al. (2018)					
		EDN3	0.85	NEGR1	0.90	Kim et al. (2014)					
				RELA	0.85	Elkrewi et al. (2024)					
	hsa-miR-3187-5p	GDNF	1.00	NEGR1	1.00	Kim et al. (2014)	GDNF	83	DAB2IP	97	Lee et al. (2012)
		L1CAM	1.00	NF2	1.00	Toledo et al. (2018)	RET	64	NRP1	92	Tillo et al. (2015)
		NRG1	1.00	DAB2IP	0.92	Lee et al. (2012)	BDNF	60	NF2	87	Toledo et al. (2018)
		RET	0.92	NFASC	0.85		NRG1	58	LRRTM3	85	
		ECE1	0.85	NSMF	0.85	Xu et al. (2010)			NSMF	75	Xu et al. (2010)
	hsa-miR-371b-5p	NRG1	1.00	DAB2IP	0.92	Lee et al. (2012)	BDNF	60	MAPK8	99	Kuil et al. (2021)
		BDNF	0.92	NF2	0.92	Toledo et al. (2018)	NRG1	58	TPM3	99	Camilleri et al. (2019)
		RET	0.92	ALKBH5	0.85	Wang et al. (2021)			NF2	98	Toledo et al. (2018)
		ECE1	0.85	MAPK8	0.85	Kuil et al. (2021)			NET1	78	Robinson et al. (2021)
				TPM3	0.85	Camilleri et al. (2019)			NCALD	74	Upadhyay et al. (2019)

Further target genes of the selected miRNA, such as HSCR-related genes or other neuronal-related genes, were searched in mRNA databases (miRWALK and miRDB).

Following that, we also searched for other genes related to critical neuronal processes for a proper Enteric Nervous System (ENS) formation, like neurogenesis, neurodevelopment or neuronal migration (see Table 3). We found genes like *ALKBH5* that could be critical for enteric neuronal crest migration in HSCR (Wang et al., 2021); in addition to others important for neuronal migration: as *NF2* (Toledo et al., 2018), *NEUROG2* (Heng et al., 2008) and *NEUROD1* (Singh et al., 2022) or *DAB2IP* (Lee et al., 2012); as well as genes participating in neuronal proliferation *NEGR1* (Kim et al., 2014), or in neuronal assembly *NFASC* (Zonta et al., 2008).

From all examined HSCR urinary downregulated miRNAs, miR-4732-5p was the one that was associated with the highest number of genes related to HSCR in both databases, 8 genes in miRWalk (*BDNF, GDNF, SOX10, L1CAM, NRG1, ECE1, ERBB3, EDN3*) together with 3 more genes in miRDB (*RET, GDNF, EDN3*); followed by hsa-miR-3187-5p with 5 genes in miRWalk (*GDNF, L1CAM, NRG1, RET, ECE1*) and 4 more in miRDB (*GDNF, RET, BDNF, NRG1*); and finally by hsa-miR-4443 with 5 genes in miRWalk (*GDNF, NRG1, ERBB3, ECE1, SOX10*) and 3 more in miRDB (*PIK3C2B, NTRK3, ECE1*) (Table 3).

Regarding the upregulated sequences, the miRNAs with more HSCR-associated gene targets were hsa-miR-210-5p with 5 genes in miRWalk (*RET, L1CAM, GDNF, BDNF, SOX10*) and 1 more in miRDB (*SOX10*); together with hsa-miR-6876-3p, also with 5 genes in miRWalk (*ERBB3, GDNF, EDNRB, ECE1, BDNF*) and one more located in miRDB (*NRG1*) (Table 3).

Altogether, the identified HSCR urinary-dysregulated miRNAs of interest targeted genes associated with HSCR pathology and also others related to neuronal processes that may be important also in ENS formation.

### 3.3 RT-qPCR validation of urinary miRNA expression

Lastly, we evaluated the expression of the above selected miRNAs (see Table 1), in the urine of the HSCR patients and controls by RT-qPCR.

**Figure 2:**
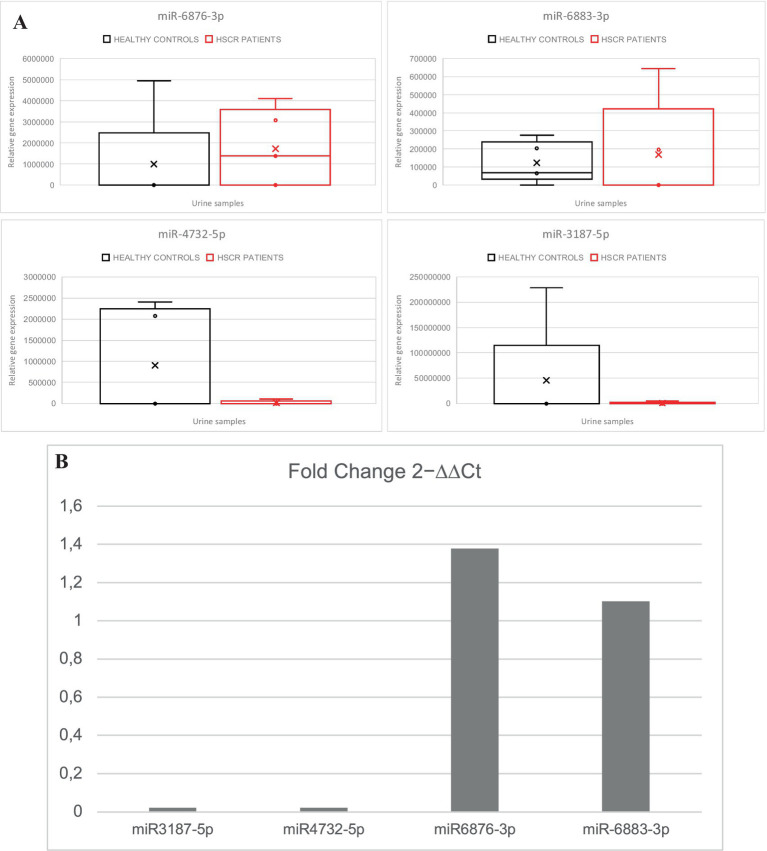
**(A)** Overview of RT-qPCR analysis of the selected miRNAs in HSCR urine samples compared to controls: miR3187-5p (F.N Test *p* = 0.000002), miR4732-5p (F.N Test *p* = 0.000015), miR6876-3p, miR-6883-3p. **(B)** Fold change comparison of 2 − ∆∆Ct from HSCR to control samples (HSCR/Controls).

Here we could verify an increased expression of hsa-miR-6883-3p and hsa-miR-6876-3p, as well as a decreased expression of hsa-miR-4732-5p (F.N Test *p* = 0.000015) and hsa-miR-3187-5p (F.N Test *p* = 0.000002) (Figure 2) in HSCR urine compared to the controls, which confirmed the previous results of those miRNAs in the microarrays.

## 4 Discussion

Our study demonstrates that specific dysregulated miRNAs can be detected in the urine of HSCR patients when compared to healthy controls. In our HSCR patients’ cohort, we could identify several upregulated and downregulated miRNAs by microarray analysis that are, related to key functional aspects in ENS development. Analyzing the predicted targets of the top 5 up and downregulated miRNAs in data bases, we could verify that they do regulate genes related to HSCR pathology, and also to neuronal processes that are maybe necessary for the proper development of the Enteric Nervous System (ENS) formation, like neurogenesis, neurodevelopment or neuronal migration (Table 3).

When we compare our results with other miRNA studies in HSCR, our identified miRNAs in HSCR urine differ from those found by others in HSCR serum (Tang et al., 2014), HSCR intestine (Shen et al., 2016; Zhang et al., 2024), or in developing ENS cells (Pai et al., 2023). However, the miRNAs found by these studies are also different between each other, possibly because of differential expression between tissue and body fluids and the specific cohorts studied.

Some of the selected miRNAs found in HSCR urine regulate *RELA* and *ERBB3* genes according to the search in miRNA databases. We recently published a downregulated expression of *ERBB3* and *RELA* mRNAs in distal aganglionic segments of HSCR patients in a cohort of 25 patients (Elkrewi et al., 2024). *ERBB3*, a member of the EGF receptor tyrosine kinase family, is known to play an important role in neural crest development (Prasad et al., 2011), while *RELA*, the main subunit of the NF-κB pathway (Perkins, 2007), participates in embryonic neurogenesis and neural progenitor migration and differentiation (Mémet, 2006; Zhang and Hu, 2012). Therefore, it could be interesting to further validate the miRNA targets at protein level in tissue or blood HSCR samples in the future.

Regarding the potential causes of miRNA dysregulation in HSCR, miRNAs expression can be regulated at transcriptional (changes in gene expression) or post-transcriptional (changes in miRNA processing) levels (Gulyaeva and Kushlinskiy, 2016). Prospective investigation is needed to assess if already known HSCR mutations mutations, or other HSCR disease’s characteristics, i.e., alterations in intestinal tissue, are somehow responsible for these miRNA modifications.

In addition, cellular pathways (Treiber et al., 2019) and various physiological and pathological stimuli, such as hormones, stress, inflammatory cytokines, DNA methylation status, etc. have been related to affect miRNA expression (Gulyaeva and Kushlinskiy, 2016; Juźwik et al., 2019). Of these, processes like inflammation, and neurodegeneration are certainly consistent with HSCR pathology and could be considered as possible miRNA dysregulation mechanisms for future investigation.

Given that our chosen list of dysregulated miRNAs in HSCR urine regulate the expression of genes related to HSCR and to neuronal and cell migration processes (see Table 3), we think they may be good candidates for HSCR biomarkers. In particular, sequences like miR-4732-5p, hsa-miR-3187-5p and hsa-miR-6876-3p target several HSCR-related genes and were validated in our RT-qPCR analysis. As an example, high levels of hsa-miR-6876-3p, which can target *GDNF, ERBB3, EDNRB* and *BDNF* mRNAs, will lead to a lower expression of those genes and thus impair neurogenesis and neuronal migration, which finally may impact the proper gut innervation in those patients.

Importantly, the expression of specific candidate miRNAs can also be detected using nanophotonic biosensors integrated into portable lab-on-a-chip platforms, which are applicable in clinical and environmental diagnostics (Tarasov et al., 2016; Smith et al., 2017; Rebelo et al., 2019; García-Chamé et al., 2020). Thus, when at some point a defined miRNA pattern would be determined, the miRNA detection could easily be implemented in a microanalytical chip system and thereafter be used routinely to complete HSCR diagnosis, potentially helping future patients.

In summary, this work provides initial results that are promising in the search of a new complementary diagnosis method for HSCR. They show they show that indeed some miRNAs have dysregulated expression as predicted. However, further research is still necessary to validate a specific miRNA signature in a larger cohort of patients that could be used as HSCR biomarker in the future. After validation, these can be used for the creation of a non-invasive, easy to interpret and relatively simple novel diagnostic tool, complementary to histology, patient history, and pathology.

This preliminary study serves as a foundation for future investigation. A larger number of patients are required to increase the robustness of the results. Ideally, patients may be recruited from a network of hospitals at national level. Once verified, selected miRNAs must be correlated with clinical history, histology and surgery outcome. Based on that, the best miRNAs may be initially added to other diagnosis approaches until their application is fully demonstrated, and finally be implemented in a regular manner.

## 5 Conclusion

HSCR patients present a detectable miRNA dysregulated signature in urine that could work as an easier, non-invasive and more affordable method to complement current Hirschsprung’s disease diagnosis tests. Therefore, further research is necessary to validate this preliminary results in a larger cohort of patients.

## Data Availability

The datasets presented in this study can be found in online repositories. The names of the repository/repositories and accession number(s) can be found at: https://www.ncbi.nlm.nih.gov/, GSE277874.

## Footnotes

^1^http://www.ncbi.nlm.nih.gov/geo/
