# Supplementary material for: Urine miRNA signature as potential non-invasive diagnostic biomarker for Hirschsprung’s disease
Source: Front Mol Neurosci. 2025 Jan 8;17:1504424. doi: 10.3389/fnmol.2024.1504424 (PMC11770682; doi:10.3389/fnmol.2024.1504424)
Supplement: Supplementary file 1 [file Table_1.docx]

## Supplementary Material

|  | **Age** | **Sex** | **Weight**  **(Kg)** | **Other**  **diseases** | **Urine collection*** | **Surgery type** | **HAEC** | **Diagnosis** | **Ileostomy** |
| --- | --- | --- | --- | --- | --- | --- | --- | --- | --- |
| **HSCR**  **Patients** | 6 m | Male | 8 | Trisomy 21 | 2 d | De la Torre | No | Hirschsprung's disease | Yes |
|  | 8 m | Male | 8 | No | 1 d | De la Torre | No | Hirschsprung's disease | Yes |
|  | 9 m | Male | 6 | Trisomy 21 | 2 d | De la Torre | No | Hirschsprung's disease | Yes |
|  | 6 y | Male | 17 | Trisomy 21 | 7 m | De la Torre | No | Hirschsprung's disease | No |
|  | 1 y | Male | 5 | No | 7 y | Colon resection | No | Jirásek-Zuelzer-Wilson-Syndrom | Yes |
| **Controls** | 10 m | Male | - | No | - | - | - | - | - |
|  | 2 y | Male | - | No | - | - | - | - | - |
|  | 4,5 y | Female | - | No | - | - | - | - | - |
|  | 6 y | Female | - | No | - | - | - | - | - |
|  | 8 y | Male | - | No | - | - | - | - | - |

**Supplementary Table 1:** Cohort characteristics. Age: m: months; y: years. *Date of urine collection, time after surgery (d: days; m: months; y: years). HAEC: Hirschsprung’s Associated Enterocolitis.

| **Name** | **Forward Sequence (5'-3')** | **Reverse Sequence (5'-3')** | **References** |
| --- | --- | --- | --- |
| **miR-4732-5p**  **MIR4732 Human qPCR Primer Pair (MI0017369)** | TGTAGAGCAGGGAGCAGGAAG | GAACATGTCTGCGTATCTC | Origene  CAT#: HP301119 |
| **miR-3187-5p**  **MIR3187 Human qPCR Primer Pair (MI0014231)** | CCTGGGCAGCGTGTGGCT | GAACATGTCTGCGTATCTC | OrigeneCAT#: HP300800 |
| **miR-371b-5p**  **MIR371b Human qPCR Primer Pair (MI0017393)** | ACTCAAAAGATGGCGGCACT | GAACATGTCTGCGTATCTC | OrigeneCAT#: HP300878 |
| **miR-378h**  **MIR378h Human qPCR Primer Pair (MI0016808)** | ACTGGACTTGGTGTCAGAT | GAACATGTCTGCGTATCTC | Origene  CAT#: HP300883 |
| **miR-210-5p** MIR210 Human qPCRPrimer Pair (MI0000286) | TGTGCGTGTGACAGCG | GAACATGTCTGCGTATCTC | Origene CAT#: HP300247 |
| **miR-6876-5p**  **MIR6876 Human qPCR Primer Pair (MI0022723)** | CAGGAAGGAGACAGGCAGTT | GAACATGTCTGCGTATCTC | OrigeneCAT#: HP301479 |
| **miR-634**  **MIR634 Human qPCR Primer Pair (MI0003649)** | AACCAGCACCCCAACTT | GAACATGTCTGCGTATCTC | Origene  CAT#: HP300545 |
| **miR-6883-3p**  **MIR6883 Human qPCR Primer Pair (MI0022730)** | AGGGAGGGTGTGGTATGGA | GAACATGTCTGCGTATCTC | Origene  CAT#: HP301492 |
| **miR-4443** | GTTGGAGGCGTGGGT | GGTCCAGTTTTTTTTTTTTTTTAAAACC | (Meerson, 2020) |
| **miR-22-3p** | GGGAAGCTGCCAGTTGAAG | GTGCGTGTCGTGGAGTCG | (Lv et al., 2018) |
| **snRNAu6** C16orf57 (USB1)Human qPCRPrimer Pair (NM_024598) | CCGTATGACCTCCTTCCACAGA | TCTGTCCACCTCTGAAACCAGG | OrigeneCAT#: HP214887 |
| **snordu6-2** | GCTTCGGCAGCACATATACTAAAAT | CGCTTCACGAATTTGCGTGTCAT |  |
| **SNORD47** | GTCGTATGCAGAGCAGGGTCCGAGGTATTCGCACT | ATCACTGTAAAACCGTTCCA | (Bayatiani et al., 2021) |

**Supplementary Table 2**: RT-qPCR primer list with sequences, ordered to OriGene Technologies GmbH, Herford, Germany.
